# Social Media Listening and Digital Profiling Study of People With Headache and Migraine: Retrospective Infodemiology Study

**DOI:** 10.2196/40461

**Published:** 2023-05-05

**Authors:** Peter Goadsby, Elena Ruiz de la Torre, Luminita Constantin, Caroline Amand

**Affiliations:** 1 NIHR King’s Clinical Research Facility King's College London London United Kingdom; 2 European Migraine and Headache Alliance Brussels Belgium; 3 Sanofi Gentilly France

**Keywords:** brand, headache, internet, migraine, social media, social support, self-management, management, digital, technology, symptoms, medicinal treatment, treatment, Twitter, blog, Youtube, drugs, ibuprofen, hydration, relaxation

## Abstract

**Background:**

There is an unmet need for a better understanding and management of headache, particularly migraine, beyond specialist centers, which may be facilitated using digital technology.

**Objective:**

The objective of this study was to identify where, when, and how people with headache and migraine describe their symptoms and the nonpharmaceutical and medicinal treatments used as indicated on social media.

**Methods:**

Social media sources, including Twitter, web-based forums, blogs, YouTube, and review sites, were searched using a predefined search string related to headache and migraine. The real-time data from social media posts were collected retrospectively for a 1-year period from January 1, 2018, to December 31, 2018 (Japan), or a 2-year period from January 1, 2017, to December 31, 2018 (Germany and France). The data were analyzed after collection, using content analysis and audience profiling.

**Results:**

A total of 3,509,828 social media posts related to headache and migraine were obtained from Japan in 1 year and 146,257 and 306,787 posts from Germany and France, respectively, in 2 years. Among social media sites, Twitter was the most used platform across these countries. Japanese sufferers used specific terminology, such as “tension headaches” or “cluster headaches” (36%), whereas French sufferers even mentioned specific migraine types, such as ocular (7%) and aura (2%). The most detailed posts on headache or migraine were from Germany. The French sufferers explicitly mentioned “headache or migraine attacks” in the “evening (41%) or morning (38%),” whereas Japanese mentioned “morning (48%) or night (27%)” and German sufferers mentioned “evening (22%) or night (41%).” The use of “generic terms” such as medicine, tablet, and pill were prevalent. The most discussed drugs were ibuprofen and naproxen combination (43%) in Japan; ibuprofen (29%) in Germany; and acetylsalicylic acid, paracetamol, and caffeine combination (75%) in France. The top 3 nonpharmaceutical treatments are hydration, caffeinated beverages, and relaxation methods. Of the sufferers, 44% were between 18 and 24 years of age.

**Conclusions:**

In this digital era, social media listening studies present an opportunity to provide unguided, self-reported, sufferers’ perceptions in the real world. The generation of social media evidence requires appropriate methodology to translate data into scientific information and relevant medical insights. This social media listening study showed country-specific differences in headache and migraine symptoms experienced and in the times of the day and treatments used. Furthermore, this study highlighted the prevalence of social media usage by younger sufferers compared to that by older sufferers.

## Introduction

Approximately 50% of people experience a headache in any given year [1,2]. Migraine is a common disabling primary headache disorder [1,3]. Migraine attacks are patient specific; few may experience increased attacks during a certain time of the year or at the end of the week or a diurnal variation in the attacks [4]. Further, migraine is differentiated into episodic migraine and chronic migraine, with episodic migraine characterized by <15 headaches per month and chronic migraine by >15 headaches per month [5]. Migraine attacks are often accompanied by nausea, vomiting, and extreme sensitivity to light and sound, which significantly affects the sufferer’s daily activities (private, social, and professional life) and overall quality of life [6-8].

Globally, migraine is the second leading cause of disability and is especially burdensome in young women, according to the Global Burden of Disease 2018 [3,9]. The prevalence of migraine has been estimated to be 6%-8% in Japan [6,10,11], 11% in Germany [12], and 14% in France [13]. Several real-world studies have attempted to provide a better understanding of migraine patterns [14-17]. The substantial burden of headache and migraine from the sufferers’ perspective and self-management approaches including nonmedication methods has been reported [14,15]. Picone et al [16] revealed that people can experience migraine symptoms for many weeks, some of which can change over time. Robblee et al [17] described the experiences of patients in whom more than 10 medications had failed and who were treated with monoclonal antibody drugs. Each of these studies has limitations, such as recall bias (retrospective self-reporting), selection bias, incorrect self-diagnosis, lost or missing data, and limited data on retrospective and prospective follow-ups as the baseline [14-17]. As headache disorders impact the community, opportunities to analyze real-world data are likely to enrich our understanding of these disorders and may aid in symptom management.

Despite the advances in treatments, the management of headache disorder is often suboptimal, generally due to misdiagnosis or underdiagnosis of the disease. Lack of training in diagnosing and managing headache disorders is also an important reason for inadequate headache health care [18,19]. Therefore, there is a growing interest in making use of technology for a better understanding and management of headache and migraine, for example, Migraine Buddy Application and Fitbit Application [14,20]. Several social media listening studies have helped strengthening and understanding patient experiences and have provided a source of real-world data, disease experiences, and health dynamics in populations, including postmarketing safety surveillance data [16,20,21]. Picone et al [16] revealed symptoms of COVID-19 not previously recognized. Powell et al [21] showed that in Twitter, more than 6 million Medical Dictionary for Regulatory Activities preferred terms [22] representing 702 individual preferred terms were discussed in the same post as a drug compared with approximately 15 million total preferred terms representing 946 individual preferred terms in Facebook [21]. It has also been shown that the distance between terms, for example, drug and disorder, could be used for identifying false positives, thereby improving adverse drug reaction detection in social media [23]. Twitter has been used to create and spread the educational content leading to significant participation by users who are unable to attend a conference and a comprehensive discussion on migraine and their experiences [24]. Patients’ experiences and emotions often shared on tweets at the onset of a migraine attack could help clinicians in determining the causes, conditions that worsen the attack, and better outcomes for patients [25,26]. YouTube users have educated themselves or others regarding the migraine symptoms, causes of pain, or its reduction [27].

Infodemiology is the science of distribution and determinant of information in an electronic medium, specifically the internet, with the ultimate aim to inform the public health and public policy [28-30]. The goal of infodemiology is leveraging the information on how, where, and when people post about their conditions. This can provide highly valuable patient-generated information, which is collected in real time. This has an advantage over traditional data collection methods, especially retrospective data collection, which might be subject to recall bias. It has been suggested that infodemiological studies help improving psychological, social, and cognitive outcomes in patients with pain [28-30]. The World Health Organization Atlas of headache disorders, worldwide, estimates around 50% of people with headache self-treated without visiting health care professionals [18]. There are limited data on the management of headache in this population. Further, lack of education is a key issue impeding good management of headache disorders. Infodemiological studies help in increasing awareness on headache and gaining insights on the perception of sufferers outside medical practice that could be included in new strategies to treat patients with pain [15,18].

We conducted an infodemiological study in Japan, Germany, and France based on social media posts on headache and migraine to identify where, when, and how people discussed their symptoms and treatments. This study aimed to capture the perspective of sufferers and to check their awareness about headache, with a unique approach of listening to their feelings and understanding their expectations. The insights from this study could be helpful for physicians and specialists to help them manage the disease better.

## Methods

### Study Design

This infodemiological study was based on the secondary use of data obtained from social media posts on headache and migraine, which were analyzed in an aggregated, cross-sectional manner at a population level. Using the Detec’t web crawler [23,31], originally developed by Kappa Santé [32], the study collected real-time data retrospectively from 5 general and specialized public social media channels (Twitter, web-based forums, blogs, review sites, and YouTube) from available posts within a 1-year period from January 1, 2018, to December 31, 2018, for Japan and a 2-year period from January 1, 2017, to December 31, 2018, for Germany and France. As data collected during 1-year period for France and Germany were less than those for Japan, the data collection period was extended by 1 year.

### Pulsar Tool

Web scraping of the messages was performed depending on the HTML structure of each channel. Searches were conducted by Pulsar company, a medical communications agency, and were based on the main language of the country being investigated.

Pulsar is the only social listening tool on the market offering social listening, monitoring, and audience segmentation in one tool. The Pulsar platform is a complete monitoring and analytics tool to measure and optimize performance across the owned social channels and websites [33]. Pulsar TRAC is linked to the Twitter application programming interface and enables researchers to scrape tweets that match specific search criteria in a bulk approach. Furthermore, Pulsar TRAC is integrated with IBM’s Watson Tone Analyzer application programming interface, a machine learning algorithm that facilitates the analysis of concepts contained in tweets [34].

### Data Collection

Posts containing at least 1 keyword (Table S1 in Multimedia Appendix 1) were automatically retrieved with all the associated metadata, deidentified, and cleaned (signature and quote withdrawal). Social listening keyword trackers collected all data publicly available on social media sites, such as Twitter (captured short-form, spontaneous opinions about headache symptoms, severity, frequency, times of the day, and treatments), web-based forums (long-form, detailed discussions about headache symptoms, severity, frequency, times of the day, and treatments), blogs (gathered individual opinions on headache symptoms, severity, frequency, and treatments), review sites (gathered opinions on treatments used; Textbox 1), and YouTube (collected opinions about headache symptoms, severity, frequency, and treatments). The total volume of posts and unique social media profiles split by social media channels are presented in Table 1. The multimedia information such as emojis, images, and videos was not managed or handled for this study.

**Most prevalent forums, blogs, and review sites.**

**Japan**

- ameblo.jp, detail.chiebukuro.yahoo.co.jp, blog.goo.ne.jp, blog.livedoor.jp, and blogs.yahoo.co.jp

**Germany**

- ubria.de, ht-mb.de, onmeda.de, jameda.de, and forum.glamour.de

**France**

- forum.doctissimo.fr, babycentre.fr, grossesse.aufeminin.com, forum.hardware.fr, and bebes.aufeminin.com

A predefined search string was used to identify social media posts and dialogues on headache and migraine in local languages. This helped in segregating data across the 3 countries. Several data aggregators with specialties across different channels and social media platforms were used to assimilate the relevant keyword-based content.

The keyword data collection used initially was selected by the authors while designing the study to reflect common terms used in the literature and in the sufferers’ real-life settings. This collection was designed with the Boolean AND operator to collect data on pain experiences (headache, migraine(s), head AND pain, and head AND sore), medicinal treatments (head AND painkiller and head AND ibuprofen), and nonpharmaceutical treatments (head AND herbal and head AND massage). Local languages, Japanese, German, and French, were used (Table S1 in Multimedia Appendix 1). Every time any of the keyword combinations were posted on social media, the study captured what was being said and the associated metadata—where and when these posts were getting published.

**Table 1:** Total volume of posts (N=3,962,872) and unique social media profiles (N=989,000) split by social media channels^a^.

Channel			Total volume of posts, n (%)		Unique social media profiles, n^b^
**Japan**					
	Twitter	2,670,525 (76)		732,000	
	Blogs	524,591 (15)		19,000	
	Forums	288,597 (8)		23,000	
	YouTube	22,698 (1)		10,000	
	Review sites	3417 (0.1)		2000	
**Germany**					
	Twitter	83,366 (57)		26,000	
	Forums	29,251 (20)		14,000	
	Blogs	14,772 (10)		6000	
	YouTube	14,480 (10)		7000	
	Review sites	4388 (3)		2000	
**France**					
	Twitter	231,532 (75)		110,000	
	Forums	53,964 (18)		25,000	
	YouTube	19,144 (6)		10,000	
	Blogs	1902 (1)		2000	
	Review sites	245 (<0.1)		200	

^a^Based on data collected from keywords.

^b^Due to anonymization of individuals, the number of unique social media profiles is presented at an aggregated level and rounded to the nearest 1000.

### Ethical Considerations

All data analyzed in this study were obtained from publicly accessible sources without accessing password-protected information. All data used within this study have been aggregated and anonymized and do not include any personally identifiable information. The use of these data for this study was legally available and ethically appropriate, as they were obtained from publicly accessible platforms and were analyzed only at a group level. All users’ personally identifiable information was strictly protected according to the user privacy terms of Twitter Inc, and all user identity-related text content from user tweets was not presented in any tables or graphics.

### Content Analysis

The key objective of the study was to collect unprompted conversational insights: conversation that was not provoked by direct questions, by identifying where, when, and how people talked about the symptoms, severity, frequency, and times of the day of their headache, medicinal products, and nonpharmaceutical treatments.

The convenience sample comprised the total headache and migraine data set to meet the primary objective.

Keyword filtering was used to derive percentage distributions of the language used in social media posts, referencing headaches and migraines. Keyword filters were then combined into broader semantic themes. The descriptive results used the volume of data for keyword filters and the percentage distributions within the respective semantic theme.

Posts refer to the number of observations. Analyses refer to the calculations performed on the total number of posts identified to segregate the number of posts describing pain (symptoms, severity, and frequency), times of the day of their headache, and use of medicinal products and nonpharmaceutical treatments.

### Audience Profiling

We considered that a method from an earlier study [35] as baseline is the common method of profiling used in social media.

Inferential analysis is essential when running infodemiology studies to provide analysis of audiences where real-world information on demographics is lacking, as individuals do not complete their social media profiles to include their age, for example, therefore ruling out descriptive statistics on the grounds of reliability.

Descriptive insights about demographic profiling of the participating audiences were carried out in an anonymized manner and aggregated format for a unique profile. The age of the sufferers was estimated by identifying specific expressions within the post. Data signals from network connectivity and URLs shared were used in the demographic profiling, a demographic analysis that is commonly used by marketers [36]. The study randomly selected sufferers by searching for keywords as described above. The age and gender were then predicted based on the language used and the screen name to define the demographic profile of the study population.

## Results

### Overview of Data

An overview of social media posts per country, posts per analyses, and unique social media profiles is shown in Table 2. A total of 3,509,828 posts were obtained from Japan in 1 year and 146,257 and 306,787 posts from Germany and France, respectively, in 2 years. Of those posts, 786,000, 55,000, and 148,000 were unique social media profiles from Japan, Germany, and France, respectively. Most sufferers discussed “headache and migraine pain experiences and symptoms” on social media (51,628 posts from Japan, 32,452 from Germany, and 25,427 from France), followed by “times of the day” (24,302 posts from Japan, 17,128 from Germany, and 28,619 from France). The most used social media platform by sufferers was Twitter (69%), followed by web-based forums (15%; Tables 1 and 2).

**Table 2:** Posts per market and posts per analyses.

Analysis			Japan (1-year period)		Germany (2-year period)		France (2-year period)
**Primary objective: to identify where, when, and how people with headache and migraine describe their symptoms and the nonpharmaceutical and medicinal treatments used as indicated on social media**							
	Total headache and migraine data set	3,509,828 posts		146,257 posts		306,787 posts	
	Pain experiences and symptoms	51,628 posts		32,452 posts		25,427 posts	
	Time of day referenced	24,302 posts		17,128 posts		28,619 posts	
	Pain experience by time of day	21,035 posts		12,597 posts		26,568 posts	
	Medicinal treatment formats	12,307 posts		17,857 posts		8257 posts	
	Medicinal treatments	4055 posts		456 posts		9691 posts	
	Nonpharmaceutical treatments	19,644 posts		11,603 posts		5026 posts	
**Secondary objective: to identify posts based on severity and frequency of symptoms**							
	Total headache and migraine data set	3,509,828 posts 786,000 unique social media profiles		146,257 posts 55,000 unique social media profiles		306,787 posts 148,000 unique social media profiles	
	Filtering by severity and frequency	153,664 posts		30,063 posts		49,088 posts	

### Demographic Profiling

Among the total unique social media profiles 989,000 noted in Table 1, only 152,044 (15.4%) posts allowed us to derive information on demographic profiling. Across the countries, the majority of the sufferers (66,844/152,044, 44%) were between 18 and 24 years of age (Table 3). The proportion of females was 63% for Japan, 51% for Germany, and 59% for France.

**Table 3:** Age distribution of the total audience.

Age (years)	Japan (N=135,000), n (%)	Germany (N=5544), n (%)	France (N=11,500), n (%)
13-17	31,050 (23)	784 (14)	1495 (13)
18-24	60,750 (45)	2184 (39)	3910 (34)
25-34	24,300 (18)	1232 (22)	3680 (32)
35-44	8100 (6)	728 (13)	1150 (10)
45-54	6750 (5)	448 (8)	920 (8)
≥55	4050 (3)	168 (3)	345 (3)

### Pain Descriptions and Symptom Dialogues

The posts and dialogues describing pain differed across countries. The lowest proportion of posts of specific pain features and symptoms associated with headache or migraine was from Japan sufferers. The most detailed posts on headache or migraine were from Germany. Japanese sufferers used specific terminology, such as either “tension headaches” or “cluster headaches” (36%), whereas French sufferers even mentioned specific migraine types, such as “ocular” (7%) and “aura” (2%). The “pain” features, in general terms, were discussed by almost one-third of the sufferers from all the countries. In Japan and France, 13% and 23% of those writing posts, respectively, mentioned “stress,” whereas “nausea,” a symptom typically associated with migraine, was discussed by 21% of those writing posts in Germany (Multimedia Appendix 2).

### Times of the Day

France was the only country where sufferers discussed more about times of the day than the pain features and symptoms (28,619 vs 24,302 posts for Japan and only 17,128 posts for Germany). The French sufferers explicitly referenced “evening” (41%) or “morning” (38%), the Japanese sufferers mentioned mainly “morning” (48%) or “night” (27%), and the German sufferers mentioned mainly “night” (41%) or “evening” (22%) times of the day (Multimedia Appendix 3).

### Pain Experience by Times of the Day

**Figure 1:**
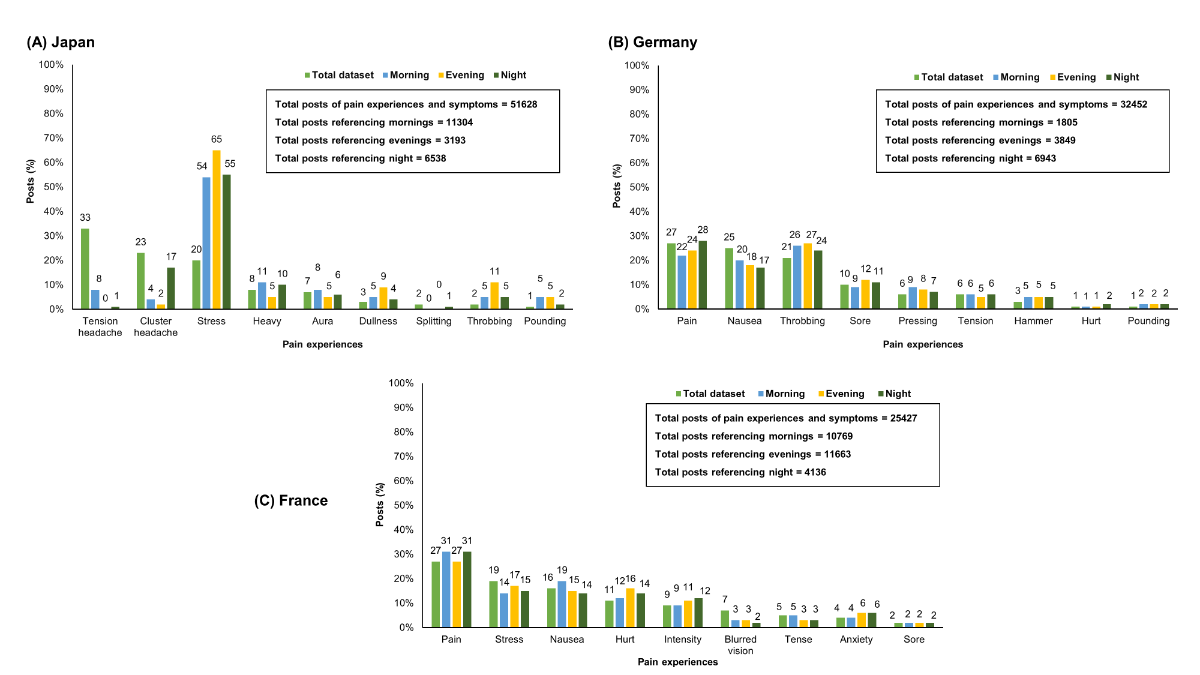
Type of pain and time of the day. (A) Japan, (B) Germany, and (C) France.

Multiple posts of pain descriptors, especially with reference to “times of the day,” were compared with the total “posts” in all countries. “Stress” was mentioned multiple times across all “times of the day” by Japan sufferers, whereas the majority of German sufferers mentioned “increased pain” as the day progressed. “Pain” was mentioned between 27% and 31% in the evening and night by France sufferers with “stress” and “anxiety” as the most prevalent pain experiences (Figure 1).

### Medicinal Treatment

The medicinal treatments were discussed mostly in generic terms in all countries. Overall, there was a preference in using generic terms rather than mentioning specific types of a drug, such as medicine, tablet, and pill painkiller (Multimedia Appendix 4). In terms of over-the-counter medicines, ibuprofen-naproxen was the most discussed combination in Japan with 43% (n=1749) of posts.

### Nonpharmaceutical Treatment

The top 3 nonpharmaceutical treatments comprised water, caffeinated beverages (coffee and tea), and relaxation methods (massages and general relaxation) across all countries. The most prevalent nonpharmaceutical treatment in Japan was staying hydrated by drinking water, which was referenced in 19% of posts, followed by massages (17%) and relaxation (8%). There was also a proclivity for using different ingredients, such as “plants” and “ginger,” to soothe the effects of headache, whereas “coffee” was discussed most frequently, with a 23% share of these posts in Germany and France (Multimedia Appendix 5).

## Discussion

### Principal Findings

This infodemiological study provided real-time, dynamic information from headache and migraine sufferers about their symptoms, severity, frequency, times of the day, and use of pharmaceutical and nonpharmaceutical treatment patterns. In this digital era, social media sites are being perceived as an instant source of information and emotional virtual support for managing headache and migraine. Many headache and migraine sufferers use social media to exchange real-life experience, to express suffering, and to vent out their frustration. An analysis of these data may improve the understanding of what is being experienced and offer insights into knowledge gaps that would help those with headache disorders, including migraine. It is somewhat surprising that despite the potential sensitivity of social media information and an increase in social media listening studies, there is a lack of national ethical guidance and harmonization on the methodology used for using social media in research [37]. However, several social media listening studies do emphasize on ethics and are transparent [16,21,38,39].

Social media listening offers a better understanding of salient migraine experiences by analyzing social media behaviors. This kind of infodemiological work could uncover conversations not conducted within the health care setting and would be helpful in better management of disease by guiding clinicians in communication with patients and in making clinical decisions. This study generated unique data from a range of social media sites, such as Twitter, web-based forums, blogs, YouTube, and review sites to obtain where, when, and how headache and migraine sufferers talked about the symptoms experienced and treatments used. Our results highlighted country-specific differences in the specific details of the symptoms of headache and migraine, times of the day, and medicinal or nonpharmaceutical treatment used. One important limitation for the scientific value in this kind of research remains the “hypothetic” prediction of the demographic indicators, which could potentially induce some bias in the interpretation of results. In practice, the representativeness of our population is not easily accessible. However, the study population was randomly selected in the connected social networkers or “sufferers.”

### Comparison With Previous Work

The findings of this study provide qualitative insights into the real-life experiences of headache and migraine, while there is currently limited published qualitative research, especially from web-based forums, blogs, YouTube, and review sites. Of note, the symptoms experienced, or treatments used, as discussed by the sufferers on social media sites, were consistent with those reported in other studies [40,41]. Many studies have shown the use of social media platforms, such as Twitter [24,25] and YouTube [27], to share their migraine experiences, which has proven to be effective in managing the condition [16,24,27]. In this study, sufferers from all 3 countries had similar preferences in terms of social media sites. They mostly used Twitter, followed by web-based forums and other social media sites. In a previous study, Pearson et al [27] studied data of 20 migraine sufferers from the United Kingdom, identified via migraine-specific charities, and performed interview-based questionnaires. The participants actively shared their experience through social media. Furthermore, they used social media to identify other migraine sufferers for sharing their migraine issues that they found hard to discuss with other people. In this study, a common finding across the countries was an apparently younger audience compared with the overall demographic of headache disorders. It has been reported that 8 of 10 internet users search health-related information and 74% of them use social media [42]. Interestingly, it has also been shown that social media use decreases with age [43,44] not only because of access or skill but also owing to health-related issues [45].

Twitter is an information carrier allowing people instant access to diverse sources. Most of the data generated in this study are captured from Twitter. In a cross-sectional analysis of Twitter activity by Callister et al [24], #migraine usage during conferences showed a significant increase from baseline in tweets. A longitudinal cohort study using data from Twitter [25] showed distinct sentimental profiles of patients reporting migraine experience. Furthermore, another infodemiological study of migraine using Twitter data, by Nascimento et al [26], was focused on word frequencies and attention to descriptions of “migraine impact,” “pain descriptors,” and geographical and temporal patterns. The study collected 21,741 migraine tweets from 64.5% of users reporting their migraine headache attacks in real time. A total of 1165 (19.9%) of these tweets came from Europe. The word “throbbing” was used in 1.5% of cases (20 uses), “pounding” in 1.2% of cases (16 uses), and “splitting” in 0.7% of cases (9 uses). In this study, there was 9.5% use of “throbbing” in Japan and Germany, and only 1% use of “pounding” and “splitting” was reported in Japan. A Google trend study on pain [39] reported “headache” as the most frequent search in 41 countries, including most of North and South America, North European countries, Turkey, South Africa, India, Japan, Australia, and New Zealand. Another observational Irish study on pain-related tweets by Mullins et al [46] identified 941 tweets from 715 contributors. The 2 most frequently occurring keywords were headache (n=321) and migraine (n=147). Most of the tweets related to headache (90%) and migraine (66%) were generated from patients’ reporting of ongoing symptoms. In our study, “headache” was the second most terminology related to pain mentioned by Japanese sufferers. In addition, they also specified “tensions” or “cluster headaches.”

Our study findings showed country-specific variations in terms of sufferers mentioning their “pain experience” and medicinal and nonpharmaceutical treatment patterns used. The findings of this study provide valuable information on web-based discussions involving “medicinal treatment terminologies” used by the sufferers allowing a better understanding of the needs and concerns of sufferers taking or considering taking headache and migraine medicines. The mention of medicines used by sufferers within this study appears to concur with previous findings. Paracetamol alone or in combination was mentioned from 81 posts—7284 posts in all countries with a higher proportion in Japan and France, which is in line with a French study that found that the majority (7.5%) of sufferers were using paracetamol alone or in combinations for managing their headache and migraine [47]. The use of analgesics for headache and migraine is very common, and many countries recommend using these as over-the-counter treatment [48-51]. In contrast to other infodemiology studies, our study gathered additional data on “nonpharmaceutical and medicinal treatments” used by the headache and migraine sufferers in all countries. These findings provide valuable insights on the power of these social media tools and the impact that they can have on treatment perception. Additionally, this study provides crucial real-time information about the sufferers’ problems and medications or nonpharmaceutical approach. Interestingly, a recent study with app data collection in Japan, Germany, and France showed similar findings. Most users reported “tension-type headache” followed by “cluster headache.” The majority of sufferers reported sleeping, drinking water, and coffee as nonpharmaceutical treatments to manage their headache and migraine [14].

The data acquired within this study give a unique insight about headache and migraine sufferers’ behavior and perspective in different countries on different social platforms and thus provide a unique way to better understand disease burden and unmet needs of these sufferers by studying real-life experiences beyond cultural aspects. The spontaneous and real-time responses captured by social media posts on the onset of a migraine attack could assist clinicians in determining what types of triggers and conditions produce worse or better outcomes for sufferers. These types of studies reveal what are the daily life experiences of sufferers, their behaviors, and perceptions outside the medical setting in the real world. Information about a patient’s emotional responses or expressive patterns related to migraine onset could potentially become an effective assessment in predicting adjustment to the migraine burden. In this study, the age and gender were predicted based on the language used and the screen name to define the demographic profile of the study population, which could have led to a bias in data interpretation. Further, it was not possible to determine age and gender for some profiles, which is one of the limitations of a real-world study. In addition, as the findings of this study show variation in terms of “pain experiences,” “times of the day,” and use of treatments across countries, these real-life expressions of headache and migraine sufferers cannot be extrapolated globally. Therefore, further research conducting similar infodemiological studies in other geographical regions is needed, which will generate extensive generalizable data and, in return, avoid reductionism.

### Conclusions

Social media listening has proven to be a powerful source of knowledge for headache and migraine research. The use of health-related social media is a growing and constantly updating source of real-world data. The perspectives generated from analyzing social media may help in conveying the unmet needs of sufferers in real time, which would help provide a comprehensive understanding of the disease impact and outcomes, including self-medication therapeutic solutions used, which otherwise is not possible to capture in traditional sources of real-world data. Outputs from health-related social media listening studies can help address future research questions and set up research hypothesis.

This study provided a robust understanding of how and when sufferers talk about their headaches or migraines in Japan, Germany, and France. The web audiences were found to be younger; therefore, younger sufferers should be considered while addressing headache sufferers on the web versus offline. This study highlighted country-specific differences. When it comes to specific details on the symptoms of headache and migraine, or times of the day, sufferers from Japan spoke the least, whereas those from Germany spoke the most. Sufferers from Europe provided more details about when their headaches were occurring. German sufferers spoke the most about nonpharmaceutical and medicinal treatments used, whereas their French counterparts spoke the most about treatments used.

## Appendix

Multimedia Appendix 1 Keywords and translation.

Multimedia Appendix 2 Most frequently mentioned terminologies related to pain experiences and symptoms. (A) Japan, (B) Germany, and (C) France.

Multimedia Appendix 3 Time of day referenced. (A) Japan, (B) Germany, and (C) France.

Multimedia Appendix 4 Medicinal treatment formats. (A) Japan, (B) Germany, and (C) France. OTC (over-the-counter).

Multimedia Appendix 5 Nonpharmaceutical treatments. Some nonpharmaceutical treatments were added later manually and were not part of the predefined list. (A) Japan, (B) Germany, and (C) France.

## Data Availability

Qualified researchers may request access to data and study-related documents. Further details on Sanofi’s data sharing criteria, eligible studies, and process for requesting access can be found at Vivli [52].

## References

[1] (2016). Headache disorders. World Health Organization.

[2] Vosoughi K, Stovner LJ, Steiner TJ, Moradi-Lakeh M, Fereshtehnejad SM, Farzadfar F, Heydarpour P, Malekzadeh R, Naghavi M, Sahraian MA, Sepanlou SG, Tehrani-Banihashemi A, Majdzadeh R, Feigin VL, Vos T, Mokdad AH, Murray CJL (2019). The burden of headache disorders in the Eastern Mediterranean Region, 1990-2016: findings from the global burden of disease study 2016. J Headache Pain.

[3] GBD 2019 DiseasesInjuries Collaborators (2020). Global burden of 369 diseases and injuries in 204 countries and territories, 1990-2019: a systematic analysis for the global burden of disease study 2019. Lancet.

[4] Poulsen AH, Younis S, Thuraiaiyah J, Ashina M (2021). The chronobiology of migraine: a systematic review. J Headache Pain.

[5] No authors listed (2018). Headache classification committee of the international headache society (IHS) the international classification of headache disorders, 3rd edition: migraine. Cephalalgia.

[6] Takeshima T, Wan Q, Zhang Y, Komori M, Stretton S, Rajan N, Treuer T, Ueda K (2019). Prevalence, burden, and clinical management of migraine in China, Japan, and South Korea: a comprehensive review of the literature. J Headache Pain.

[7] Martelletti P, Schwedt TJ, Lanteri-Minet M, Quintana R, Carboni V, Diener HC, Ruiz de la Torre E, Craven A, Rasmussen AV, Evans S, Laflamme AK, Fink R, Walsh D, Dumas P, Vo P (2018). My migraine voice survey: a global study of disease burden among individuals with migraine for whom preventive treatments have failed. J Headache Pain.

[8] Ueda K, Ye W, Lombard L, Kuga A, Kim Y, Cotton S, Jackson J, Treuer T (2019). Real-world treatment patterns and patient-reported outcomes in episodic and chronic migraine in Japan: analysis of data from the Adelphi migraine disease specific programme. J Headache Pain.

[9] Steiner TJ, Stovner LJ, Jensen R, Uluduz D, Katsarava Z, Lifting The Burden: the Global Campaign against Headache (2020). Migraine remains second among the world's causes of disability, and first among young women: findings from GBD2019. J Headache Pain.

[10] Kikui S, Chen Y, Todaka H, Asao K, Adachi K, Takeshima T (2020). Burden of migraine among Japanese patients: a cross-sectional National Health and Wellness Survey. J Headache Pain.

[11] Sakai F, Igarashi H (1997). Prevalence of migraine in Japan: a nationwide survey. Cephalalgia.

[12] Roessler T, Zschocke J, Roehrig A, Friedrichs M, Friedel H, Katsarava Z (2020). Administrative prevalence and incidence, characteristics and prescription patterns of patients with migraine in Germany: a retrospective claims data analysis. J Headache Pain.

[13] Demarquay G, Moisset X, Lantéri-Minet M, de Gaalon S, Donnet A, Giraud P, Guégan-Massardier E, Lucas C, Mawet J, Roos C, Valade D, Ducros A (2021). Revised guidelines of the French headache society for the diagnosis and management of migraine in adults. Part 1: diagnosis and assessment. Rev Neurol (Paris).

[14] Goadsby PJ, Constantin L, Ebel-Bitoun C, Igracki Turudic I, Hitier S, Amand-Bourdon C, Stewart A (2021). Multinational descriptive analysis of the real-world burden of headache using the migraine buddy application. Eur J Neurol.

[15] Goadsby PJ, Lantéri-Minet M, Michel MC, Peres M, Shibata M, Straube A, Wijeratne T, Ebel-Bitoun C, Constantin L, Hitier S (2021). 21st century headache: mapping new territory. J Headache Pain.

[16] Picone M, Inoue S, DeFelice C, Naujokas MF, Sinrod J, Cruz VA, Stapleton J, Sinrod E, Diebel SE, Wassman ER (2020). Social listening as a rapid approach to collecting and analyzing COVID-19 symptoms and disease natural histories reported by large numbers of individuals. Popul Health Manag.

[17] Robblee J, Devick KL, Mendez N, Potter J, Slonaker J, Starling AJ (2020). Real-world patient experience with erenumab for the preventive treatment of migraine. Headache.

[18] World Health Organisation (2011). Atlas of Headache Disorders and Resources in the World.

[19] Steiner TJ, Antonaci F, Jensen R, Lainez MJA, Lanteri-Minet M, Valade D, European Headache Federation, Global Campaign againist Headache (2011). Recommendations for headache service organisation and delivery in Europe. J Headache Pain.

[20] Cerrada CJ, Min JS, Constantin L, Hitier S, Igracki Turudic I, Amand-Bourdon C, Stewart A, Ebel-Bitoun C, Goadsby PJ (2022). A prospective real-world study exploring associations between passively collected tracker data and headache burden among individuals with tension-type headache and migraine. Pain Ther.

[21] Powell GE, Seifert HA, Reblin T, Burstein PJ, Blowers J, Menius JA, Painter JL, Thomas M, Pierce CE, Rodriguez HW, Brownstein JS, Freifeld CC, Bell HG, Dasgupta N (2016). Social media listening for routine post-marketing safety surveillance. Drug Saf.

[22] International Council for Harmonisation of Technical Requirements for Pharmaceuticals for Human Use (2021). Medical Dictionary for Regulatory Activities version 24.0. MedDRA.

[23] Abdellaoui R, Schück S, Texier N, Burgun A (2017). Filtering entities to optimize identification of adverse drug reaction from social media: how can the number of words between entities in the messages help?. JMIR Public Health Surveill.

[24] Callister MN, Robbins MS, Callister NR, Vargas BB (2019). Tweeting the headache meetings: cross-sectional analysis of twitter activity surrounding American headache society conferences. Headache.

[25] Deng H, Wang Q, Turner DP, Sexton KE, Burns SM, Eikermann M, Liu D, Cheng D, Houle TT (2020). Sentiment analysis of real-world migraine tweets for population research. Cephalalgia Reports.

[26] Nascimento TD, DosSantos MF, Danciu T, DeBoer M, van Holsbeeck H, Lucas SR, Aiello C, Khatib L, Bender MA, Zubieta JK, DaSilva AF, UMSoD (Under)Graduate Class Of 2014 (2014). Real-time sharing and expression of migraine headache suffering on Twitter: a cross-sectional infodemiology study. J Med Internet Res.

[27] Pearson C, Swindale R, Keighley P, McKinlay AR, Ridsdale L (2019). Not just a headache: qualitative study about web-based self-presentation and social media use by people with migraine. J Med Internet Res.

[28] Eysenbach G (2002). Infodemiology: the epidemiology of (mis)information. Am J Med.

[29] Eysenbach G (2009). Infodemiology and infoveillance: framework for an emerging set of public health informatics methods to analyze search, communication and publication behavior on the internet. J Med Internet Res.

[30] Eysenbach G (2011). Infodemiology and infoveillance tracking online health information and cyberbehavior for public health. Am J Prev Med.

[31] Kürzinger ML, Schück S, Texier N, Abdellaoui R, Faviez C, Pouget J, Zhang L, Tcherny-Lessenot S, Lin S, Juhaeri J (2018). Web-based signal detection using medical forums data in France: comparative analysis. J Med Internet Res.

[32] (2023). CRO Specialist in real world evidence. Kappa Santé.

[33] (2022). Social listening has evolved. Pulsar.

[34] Gerrath MHEE, Mafael A, Ulqinaku A, Biraglia A (2023). Service failures in times of crisis: an analysis of eWOM emotionality. J Bus Res.

[35] Bayot RK, Gonçalves T (2018). Age and gender classification of tweets using convolutional neural networks.

[36] Chelba C, Mikolov T, Schuster M, Ge Q, Brants T (2014). One billion word benchmark for measuring progress in statistical language modeling. https://arxiv.org/abs/1312.3005.

[37] Hunter RF, Gough A, O'Kane N, McKeown G, Fitzpatrick A, Walker T, McKinley M, Lee M, Kee F (2018). Ethical issues in social media research for public health. Am J Public Health.

[38] Kondziella D, Olsen MH, Dreier JP (2020). Prevalence of visual snow syndrome in the UK. Eur J Neurol.

[39] Kamiński M, Łoniewski I, Marlicz W (2020). "Dr. Google, i am in pain"-global internet searches associated with pain: a retrospective analysis of google trends data. Int J Environ Res Public Health.

[40] Hirata K, Ueda K, Ye W, Kim Y, Komori M, Jackson J, Cotton S, Rajan N, Treuer T (2020). Factors associated with insufficient response to acute treatment of migraine in Japan: analysis of real-world data from the adelphi migraine disease specific programme. BMC Neurol.

[41] Lombard L, Ye W, Nichols R, Jackson J, Cotton S, Joshi S (2020). A real-world analysis of patient characteristics, treatment patterns, and level of impairment in patients with migraine who are insufficient responders vs responders to acute treatment. Headache.

[42] Ventola CL (2014). Social media and health care professionals: benefits, risks, and best practices. P T.

[43] Cotten SR, Schuster AM, Seifert A (2022). Social media use and well-being among older adults. Curr Opin Psychol.

[44] Merkel S, Hess M (2020). The use of internet-based health and care services by elderly people in Europe and the importance of the country context: multilevel study. JMIR Aging.

[45] Ang S, Lim E, Malhotra R (2021). Health-related difficulty in internet use among older adults: correlates and mediation of its association with quality of life through social support networks. Gerontologist.

[46] Mullins CF, Ffrench-O'Carroll R, Lane J, O'Connor T (2020). Sharing the pain: an observational analysis of Twitter and pain in Ireland. Reg Anesth Pain Med.

[47] Schück S, Roustamal A, Gedik A, Voillot P, Foulquié P, Penfornis C, Job B (2021). Assessing patient perceptions and experiences of paracetamol in France: infodemiology study using social media data mining. J Med Internet Res.

[48] Müller B, Dresler T, Gaul C, Glass A, Jürgens TP, Kropp P, Ruscheweyh R, Straube A, Förderreuther S (2019). More attacks and analgesic use in old age: self-reported headache across the lifespan in a German sample. Front Neurol.

[49] Donnet A, Emery C, Aly S, Allaf B, Cayre F, Mahieu N, Gourmelen J, Levy P, Fagnani F (2019). Migraine burden and costs in France: a nationwide claims database analysis of triptan users. J Med Econ.

[50] Meyers JL, Davis KL, Lenz RA, Sakai F, Xue F (2019). Treatment patterns and characteristics of patients with migraine in Japan: a retrospective analysis of health insurance claims data. Cephalalgia.

[51] Diener HC, Tassorelli C, Dodick DW, Silberstein SD, Lipton RB, Ashina M, Becker WJ, Ferrari MD, Goadsby PJ, Pozo-Rosich P, Wang SJ, Mandrekar J, International Headache Society Clinical Trials Standing Committee (2019). Guidelines of the international headache society for controlled trials of acute treatment of migraine attacks in adults: fourth edition. Cephalalgia.

[52] Vivli.

