# Supplementary material for: Social Media Listening and Digital Profiling Study of People With Headache and Migraine: Retrospective Infodemiology Study
Source: J Med Internet Res. 2023 May 5;25:e40461. doi: 10.2196/40461 (PMC10199393; doi:10.2196/40461)
Supplement: Multimedia Appendix 1 [file jmir_v25i1e40461_app1.docx]

**Supplementary Table S1.** Keywords and translation

| **Keywords** | **Japanese translation** | **German translation** | **French translation** |
| --- | --- | --- | --- |
| Entire headache and migraine dataset | | | |
| Headache^a^ | 頭痛 / 頭痛い / 頭が痛い | Kopfweh / Kopfschmerzen / Kopfschmerz  / Kopf schmerzend | mal de tête / maux de tête / mal à la tête / maux de tête / tête fait mal / tête faisant mal / Céphalée / céphalées |
| Migraine^a^ | 片頭痛 / 偏頭痛 | Migräne / Migränen | migraine / migraines |
| Head^b^ | 頭 / あたま | Kopf / Köpfe / Schädel | tête / têtes |
| Pain | 痛 | Schmerz | Maux / Douloureux / Douloureuse / Douloureusement |
| Pain | 痛み | NA | NA |
| Pains | NA | Schmerzen | NA |
| Painful | 痛い | schmerzhaft | NA |
| Painfully | 痛くて | schmerzlich | NA |
| Hurt | NA | schmerzt / weh tun | faire du mal / fait du mal / avoir mal / j'ai mal / |
| Hurts | NA | tut weh | NA |
| Sore | ヒリヒリ | Weh | Endolori / endolorissement |
| Soreness | ヒリヒリする | NA |  |
| Tense | 張る | NA | Tendue / tension |
| Tension | 張り | Spannungsschmerz / angespannt / spannen | NA |
| Pounding | ガンガン | Pochen | Martèlement |
| Throbbing | ズキズキ | Ziehen | Battante / lancinante |
| Pulsating | NA |  | Pulsatile |
| Pressing | NA | Drücken | NA |
| Stinging | NA | Stechen | NA |
| Hammering | NA | Hämmern | NA |
| Tense | 締め付け | NA | NA |
| Ache | 疼き | NA | NA |
| Aches | 疼く | NA | NA |
| Aching | 疼痛 | NA | NA |
| Pain | いたみ | NA | NA |
| Hurts | いたい | NA | NA |
| Painfully | いたくて | NA | NA |
| Painful | イタイ | NA | NA |
| Pain experiences | | | |
| Pain | 痛み / 痛い / 痛む | Schmerz / Schmerzen / schmerzhaft / schmerzlich / schmerzt | douleur / douloureuse / douloureuses |
| Hurt | NA | NA | Souffrance |
| Intensity | NA | NA | intense / intenses / insupportable |
| Throbbing | ズキズキ / ずきずき / ズキンズキン | ziehen / zieht / zerrt | NA |
| Nausea | NA | Übelkeit / Erbrechen / übel / mir ist schlecht / kotzen | nausée / nauséeuse / nauséeux / vomir / vomissement |
| Motion | NA | Schlimmer bei Bewegung / Schwindel | NA |
| Sore | NA | weh / wund | Endolori / endolorissement |
| Pressing | NA | drücken / drückt | NA |
| Tension | NA | Spannungsschmerz / angespannt / Kopfspannungsschmerz / spannen / Verspannungen / verspannt / Spannungskopfschmerz | Tension / Pression |
| Aura | NA | Aura | NA |
| Hammer | NA | Hämmern / hämmert / dumpf / schlagend / Schlag | NA |
| Blurred vision | 目がかすむ / かすみ目 | Sehstörungen / schlechte Augen / sehe schlecht / verschwommen / Sehnerv / "Sehbeeinträchtigung“ | Troubles visuels / points lumineux / vision déformée / troubles de la vision / troubles de la vue / migraine ophtalmique / ophtalmo / stocome |
| Heavy | 重い | NA | NA |
| Sharp pain | 激痛 | NA | NA |
| Splitting pain | 割れるように / 割れそう / 割れる | NA | NA |
| Cluster headache | 群発頭痛 | NA | NA |
| Tension headache | 緊張性頭痛 | NA | NA |
| Stress | ストレス | NA | stress / stressé / stressée |
| Dull pain | 鈍痛 / ずーん / ズーン | NA | NA |
| Pounding | ガンガン / がんがん | NA | NA |
| Back of the eyes | 目の奥 | NA | NA |
| Anxiety | NA | NA | anxiété / anxieux / angoisse |
| Medical treatment formats | | | |
| Painkiller | 痛み止め / ペインキラー | Schmerzmittel / Schmerz mittel / Schmerzstillendes Mittel / Schmerztablette / Schmerztabletten | antidouleur / antidouleurs / anti-douleur / anti-douleurs |
| Medicine | 薬 / 頭痛薬 / おくすり / お薬 | Medikament / Medikamente / Arznei / Arzneimittel / Arzneistoff | médicament / medicament / medicaments |
| Pill | NA | NA | Pilule / cachet / pastille |
| Paracetamol | パラセタモール | Paracetamol / paracetamol | paracetamol / paracetamol |
| Aspirin | アスピリン | Aspirin / aspirine / Acetylsalicylsäure / ASS | NA |
| Ibuprofen | イブプロフェン | NA | Ibuprofen / ibuprofène |
| Analgesic | 鎮痛剤 | NA | NA |
| Triptan | トリプタン | NA | NA |
| OTC medicine | 市販薬 | NA | NA |
| Capsule | カプセル | NA | Capsule / Capsules / comprimé / comprimés |
| Anti-inflammatory | 抗炎症薬 / 消炎剤 | NA | NA |
| Acetaminophen | アセトアミノフェン | NA | NA |
| Tablet | タブレット / 錠剤 | Tablette / Tabletten | NA |
| Codeine | NA | NA | codeine / codeine |
| Non-pharmaceutical treatments | | | |
| Meditation | NA | NA | méditation / méditer / médite |
| Message | NA | NA | massage / massages / acupression |
| Natural remedies | NA | NA | remède naturel / remèdes naturels / homéopathie / naturapathie / homeopathie |
| Caffeine | NA | NA | caféine / caffeine |
| Herbal | NA | NA | plante / plantes / camomille / herbes / herbal |
| Water | 水分 / (水 AND 飲む) | NA | NA |
| Massage | マッサージ | NA | NA |
| Relaxation | リラックス / リラクセーション | Entspannung / entspannend / autogenes Training / entspannen | relaxation / exercice de respiration / exercices de respiration |
| Coffee | コーヒー | Koffein / Kaffee | NA |
| Tea | お茶 / ティー / 紅茶 | Kräutertee / Tee | NA |
| Supplement | サプリ / サプリメント | NA | NA |
| Herbal | ハーブ / 漢方 | NA | NA |
| Acupuncture | 鍼 | Akkupunktur / Akupunktur / Akupunkturpunkte / Akkupunkturpunkte / Akupunkteur | NA |
| Homeopathy | NA | Homöopathie / homöopathisch / Heilpraktiker / Globuli | NA |
| Yoga | NA | Yoga | NA |
| Massage | NA | Massage / massieren | NA |
| Fresh air | NA | frische Luft / spazieren gehen | NA |
| Patches | NA | Pflaster / Heilpflaster / Wärmepflaster | NA |

Abbreviations: NA, not applicable; OTC, over-the-counter

^a^Stand-alone term.

^b^“Head” was paired with all terms below this in the list using the Boolean AND operator
(eg, “head” AND “pain”).

^c^For terms where multiple translations were applicable, the Boolean OR operator was included, so data would be collected mentioning either of the keyword translations.
