# Supplementary figures and images for: Social Media Listening and Digital Profiling Study of People With Headache and Migraine: Retrospective Infodemiology Study
Source: J Med Internet Res. 2023 May 5;25:e40461. doi: 10.2196/40461 (PMC10199393; doi:10.2196/40461)

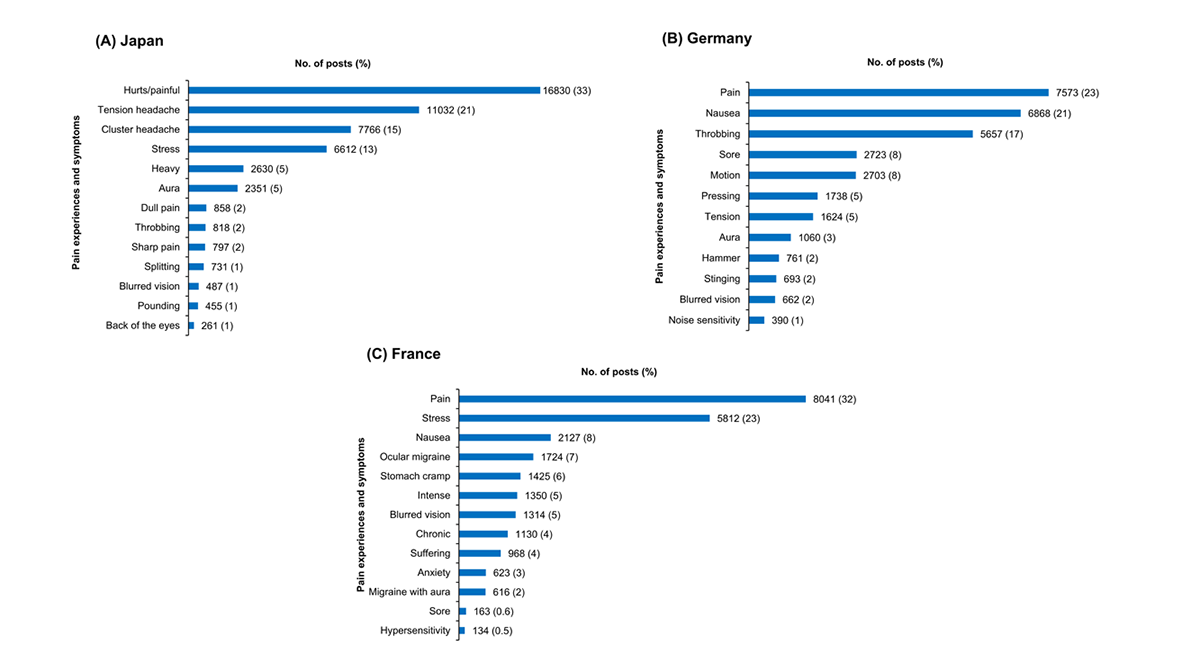

Supplement: Multimedia Appendix 2 [file jmir_v25i1e40461_app2.png]

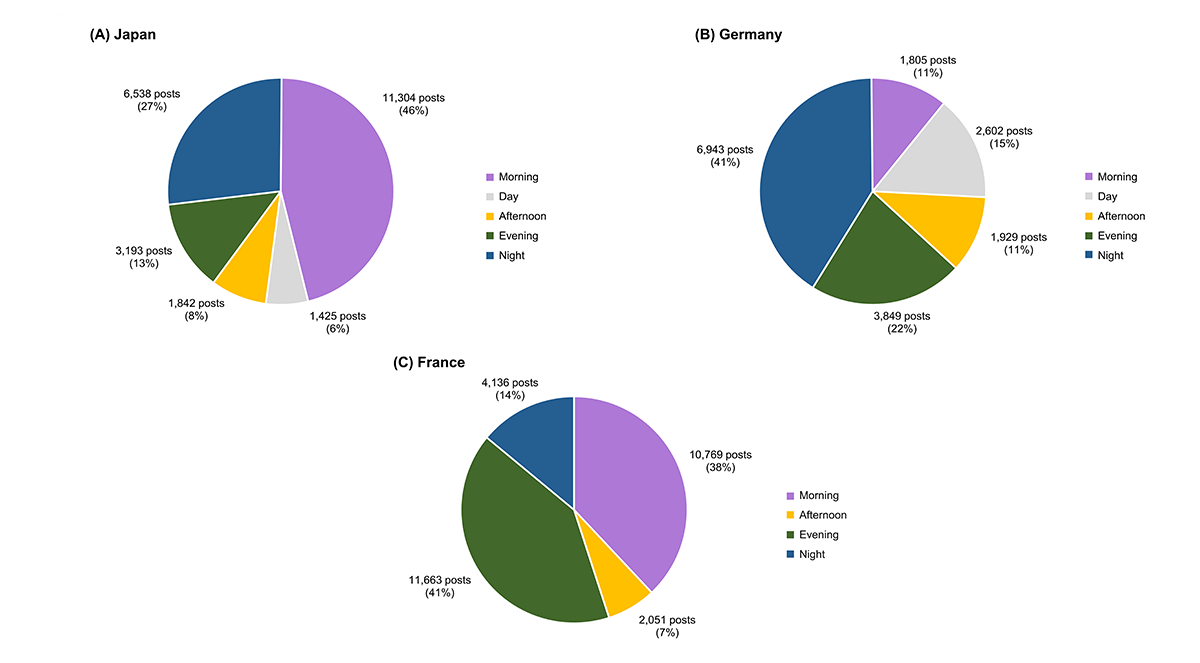

Supplement: Multimedia Appendix 3 [file jmir_v25i1e40461_app3.png]

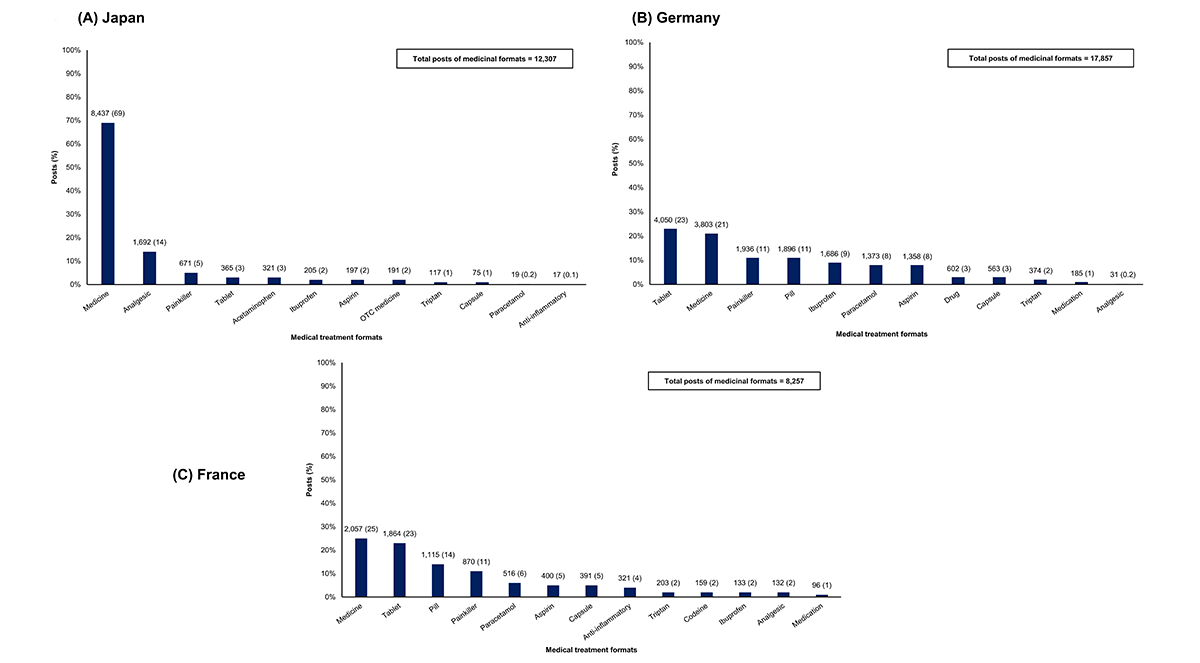

Supplement: Multimedia Appendix 4 [file jmir_v25i1e40461_app4.png]

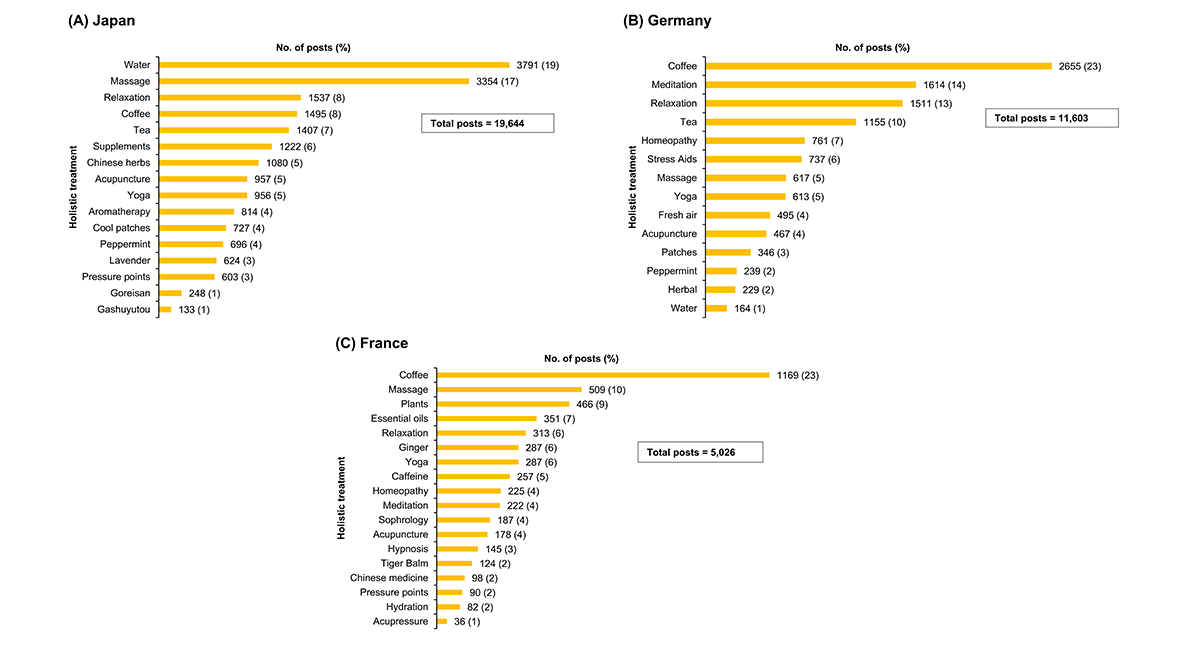

Supplement: Multimedia Appendix 5 [file jmir_v25i1e40461_app5.png]
